# Editorial: Purinergic Signaling in Health and Disease

**DOI:** 10.3389/fncel.2020.00015

**Published:** 2020-02-04

**Authors:** Eric Boué-Grabot, David Blum, Stefania Ceruti

**Affiliations:** ^1^Univ. de Bordeaux, Institut des Maladies Neurodégénératives, UMR 5293, Bordeaux, France; ^2^CNRS, Institut des Maladies Neurodégénératives, UMR 5293, Bordeaux, France; ^3^Université de Lille, Inserm, CHU, UMR-S1172, Lille Neuroscience & Cognition, Lille, France; ^4^Department of Pharmacological and Biomolecular Sciences, Università Degli Studi di Milano, Milan, Italy

**Keywords:** purine, P2X, P2Y, adenosine (A(1), A(2A), A(2B)) receptors, purinergic signaling, CNS, CNS—disorder

Adenosine 5′-triphosphate (ATP) is one of the most abundant molecules in living cells serving as universal energy “currency.” After slow acceptance of the concept of the release and extracellular action of ATP and its breakdown products, ADP and adenosine, purinergic signaling has been recognized as a widespread mechanism for cell-to-cell communication in living organisms. Additionally, the contribution of pyrimidine nucleotides (such as UTP and UDP) and sugar-nucleotides (i.e., UDP-glucose and UDP-galactose) have been more recently discovered. Purinergic signaling plays major physiological roles in mammalian central nervous system (CNS) such as neurotransmission, neuromodulation, communication in glial network, and between neurons and glia. The high number of signaling molecules provides the versatile basis for complex purinergic signaling through the activation of several families of receptors. G-protein coupled P1 receptors for adenosine, ionotropic P2X receptors for ATP and G-protein coupled P2Y receptors for ATP and other nucleotides are abundant and widely distributed in central neurons at pre-and post-synapse and in glial cells. Dysregulations of purinergic signals are associated with major CNS disorders including chronic pain, brain trauma ischemia, epilepsy, neurodegenerative diseases such as Alzheimer's disease (AD) or Amyotrophic lateral sclerosis (ALS) associated with neuro-inflammation as well as neuropsychiatric diseases, including depression, anxiety, and schizophrenia.

In this Research Topic we have brought together 22 articles written by 145 authors containing 7 reviews, 1 hypothesis and theory, 1 brief research report, and 13 original research articles. Review articles present several up-to-date aspects of the biology of purinergic signaling in the nervous system, such as in the vertebrate olfactory system (Rotermund et al.) or in the preBötzinger Complex (Reklow et al.). Other reviews focus on the structure-function of P2X receptors (Peverini et al.) and on the function of adenosine receptor agonists, guanine-based purines and vesicular nucleotide transporter in health and disease emphasizing their therapeutic potential in neurological disorders (Jacobson et al.; Tasca et al.; Miras-Portugal et al.). The function of P2X7 receptor and its regulation by its wide interactome is also reviewed (Kanellopoulos and Delarasse; Kopp et al.). In addition, the diversity of P2X7 function is underlined in an original research article showing the role of P2X7 in the regulation of the whole-body energy metabolism (Giacovazzo et al.). P2X7 receptor distribution was also examined in a ß-amyloid mouse model and revealed its microglial upregulation at advanced and late stages of the disease (Martínez-Frailes et al.). The other original research papers are covering important aspects of purinergic receptor function and regulation in the CNS. Several papers focus on the role of adenosine A_2A_ receptors expressed in distinct brain region such as the prefrontal cortex (PFC), the hippocampus and the striatum and their role of associated behavior. By a selective downregulation of A_2A_R selectively in prelimbic medial PFC the authors revealed the role of A_2A_ in physiological behaviors such as decision making (Leffa et al.). A distinct strategy was used to knockdown A_2A_R in two striatal regions, the nucleus accumbens and the dorsal medium striatum and results show that downregulation of A_2A_ increased attention and motivation (Zhou et al.). Long lasting blockade of A_2A_ receptor activity by a selective antagonist in a ß-amyloid mouse model of AD improves memory deficit and reduces cortical amyloid load consistent with a beneficial role of A_2A_R blockade for AD (Faivre et al.) and with the idea that consumption of caffeine reduces the risk of developing the pathology. However, an article reveals that caffeine consumption during pregnancy may have opposite effect representing a risk-factor for early appearance of AD symptoms in the off-springs of a transgenic mouse model of tauopathy (Zappettini et al.). Several articles focus on P2X4 receptors describing new structural features based on the identification of a selective allosteric inhibitory site (Ase et al.) or novel specific monoclonal antibodies and nanobodies recognizing specifically P2X4 receptors in its native conformation (Bergmann et al.), as well as a role of intracellular P2X4 in lysosomal exocytosis that may be implicated in HIV associated neuropathy (Datta et al.). In the striatum, pharmacological manipulations of P2X4 and dopamine receptors show the role of the interplay between purinergic and dopaminergic signaling in the regulation of sensorimotor information processing (Khoja et al.). Spatio-temporal expression profile of P2X subunits during embryogenesis in *Xenopus* reveal that several P2X receptors may have distinct role during development including neurogenesis (Blanchard et al.). Neuromodulation of cortical excitatory or inhibitory synapses by P2X receptors activated by ATP released from neurons as well as from astrocytes in the neocortex is altered during aging, suggesting that alteration of ATP signaling may contribute to age-dependent impairment of synaptic activities, but can be partially rescued by positive experience, such as environmental enrichment (Lalo et al.). Fasting/refeeding conditions in rats show that increased expression of P2X2 is associated to increase neuronal activity in the supraoptic nucleus of the hypothalamus and arginine vasopressin release (Ivetic et al.). Finally, purinergic profiling of blood T cells of patients with migraine suggests the involvement of purinergic signaling in this disorder with increase of ATP-dependent pro-inflammatory and reduction of adenosine-mediated anti-inflammatory mechanism (Nurkhametova et al.).

Overall, this Research Topic provides an up-to-date overview of the diversity of the purinergic signaling function in the CNS and provides new insights on the importance of their engagement in physiological and pathological conditions.

## Author Contributions

All authors listed have made a substantial, direct and intellectual contribution to the work, and approved it for publication.

### Conflict of Interest

The authors declare that the research was conducted in the absence of any commercial or financial relationships that could be construed as a potential conflict of interest.

